# A Disintegrin and Metalloproteinase (ADAM) Family: Their Significance in Malignant Tumors of the Central Nervous System (CNS)

**DOI:** 10.3390/ijms221910378

**Published:** 2021-09-26

**Authors:** Marta Łukaszewicz-Zając, Maciej Dulewicz, Barbara Mroczko

**Affiliations:** 1 Department of Biochemical Diagnostics, Medical University, 15-269 Bialystok, Poland; mroczko@umb.edu.pl; 2 Department of Neurodegeneration Diagnostics, Medical University, 15-269 Bialystok, Poland; maciejdulewicz@gmail.com

**Keywords:** ADAM, ADAMTS, biomarker, central nervous system, tumor

## Abstract

Despite the considerable advances in diagnostic methods in medicine, central nervous system (CNS) tumors, particularly the most common ones—gliomas—remain incurable, with similar incidence rates and mortality. A growing body of literature has revealed that degradation of the extracellular matrix by matrix metalloproteinases (MMPs) might be involved in the pathogenesis of CNS tumors. However, the subfamily of MMPs, known as disintegrin and metalloproteinase (ADAM) proteins are unique due to both adhesive and proteolytic activities. The objective of our review is to present the role of ADAMs in CNS tumors, particularly their involvement in the development of malignant gliomas. Moreover, we focus on the diagnostic and prognostic significance of selected ADAMs in patients with these neoplasms. It has been proven that ADAM12, ADAMTS4 and 5 are implicated in the proliferation and invasion of glioma cells. In addition, ADAM8 and ADAM19 are correlated with the invasive activity of glioma cells and unfavorable survival, while ADAM9, -10 and -17 are associated with tumor grade and histological type of gliomas and can be used as prognostic factors. In conclusion, several ADAMs might serve as potential diagnostic and prognostic biomarkers as well as therapeutic targets for malignant CNS tumors. However, future research on ADAMs biology should be performed to elucidate new strategies for tumor diagnosis and treatment of patients with these malignancies.

## 1. Malignant Tumors of the Central Nervous System (CNS)—General Characteristics

Primary tumors of the central nervous system (CNS) represent approximately 2% of all cancer cases worldwide. These tumors belong to a highly heterogeneous group of neoplasms with variable disease courses and different patient prognoses. Brain tumors comprise different types of primary brain tumors and a variety of secondary neoplasms [1,2]. Primary CNS tumors represent a range of low as well as high-grade neoplasms. Based on the World Health Organization (WHO) classification, the histological grading system of CNS tumors is presented as grades–from I to IV, where grades I and II are low-grade tumors, whereas grades III and IV are classified as high-grade neoplasms [1,2]. CNS tumors are also divided into categories, including tumors of the cranial and paraspinal nerves, and neuroepithelial tissue, tumors of the meninges and the sellar region, lymphomas and hematopoietic neoplasms as well as germ cell and metastatic tumors, while primary brain tumors are classified based on cellular origin and histological appearance [3]. It has been estimated that the most common malignancy of CNS are tumors of neuroepithelial origin–gliomas, which account for over 40 percent of all primary malignant CNS tumors [1,2].

Gliomas occur primarily in the brain and are derived from the cellular elements of the glia, i.e., astrocytes and oligodendrocytes. Meningiomas arise from meningothelial cells and account for approximately 30% of all malignant CNS neoplasms. In addition, low-grade infiltrating gliomas (LGG) of the cerebral hemispheres are currently classified as grade II tumors and include oligoastrocytomas, astrocytomas and oligodendrogliomas. They often develop in young patients and are characterized by longer-term survival in comparison with high-grade gliomas (HGG). However, around 50 percent of grade II gliomas may progress to high-grade gliomas within five years [4,5]. In addition, grade III glial tumors consist of anaplastic astrocytoma, mixed anaplastic oligoastrocytoma and anaplastic oligodendroglioma, whereas grade IV represents glioblastoma (GBM) which accounts for approximately 55% of all neuroepithelial tumors in adult patients [6].

There are two different types of GBM, primary and secondary. Around 90% of all GBM occur as primary GBM (PrGBM) and develop de novo, without a pre-existing lower-grade glioma. CNS tumors of this type are common in patients aged over 60 years. Approximately 10% of CNS tumor cases are secondary GBM tumors (ScGBM) that commonly arise from pre-existing tumors, such as grade II or III astrocytomas or mixed gliomas, and are typical of patients younger than 45 years [4,5]. Both types of GBM have similar histology, but are characterized by distinct genetic alterations, such as mutations in isocitrate dehydrogenase (IDH) or 19q and 22q loss of heterozygosity [7,8,9]. Therefore, these tumors should be considered as two different diseases [10].

Unfortunately, the etiology of CNS tumors, particularly GBM, is still poorly understood. However, it has been reported that genetic alterations such as mutations, loss of telomerase, activation of oncogenes and induction of aneuploidy as well as epigenetic and molecular changes play a potential role in the development of CNS tumor, including gliomas [2,11,12,13]. Epigenetic modifications such as DNA and RNA methylation as well as histone modifications have been proven to participate in the cancer stem cells (CSCs) formation. Therefore, some authors suggest that identifying CSCs and targeting epigenetic pathways may offer new insights into the treatment of cancer [14]. In addition, epigenetic mechanisms might play a role in CNS tumors via synthesis and deposition of collagens into the brain parenchyma combined with enhanced MMPs activity, which can facilitate rapid invasion of tumor cells through the brain. Therefore, it is suggested that the epigenetic mechanisms which control MMPs, TIMPs and collagens activities may represent promising drug targets in the future [14]. Additionally, gliomas are able to induce changes in normal gene expression without altering the DNA sequence. Aberrant epigenetic mechanisms, including histone modifications, DNA methylation, chromatin remodelling or altered noncoding RNA expression have been proven to be relevant in the process of CNS tumor formation. The majority of investigations have focused on DNA methylation, including hypermethylation of CpG islands which is associated with tumor-suppressor gene silencing. In addition, gene-specific hypomethylation, resulting in aberrant activation of oncogenes as well as genome-wide hypomethylation are also indicated as epigenetic changes in GBM. Furthermore, multiple alterations in the DNA methylation pattern of promoters of genes involved in cell cycle regulation, such as CDKN2A-p16INK4a, tumor suppression including RB, VHL or epithelial membrane protein 3 (EMP3), DNA repair and genome integrity (e.g., MGMT and hMLH1) as well as genes associated with the regulation of tumor invasion and inhibition of apoptosis, such as DAPK1, TIMP3, CDH1, PCDHGA11 and TMS1/ASC have been also found as epigenetic alterations presented in gliomas [15,16]. Moreover, various environmental risk factors, including alcohol and tobacco use, mobile phone use, exposure to chemical agents (pesticides), head trauma, injury, exposure to low-frequency electromagnetic fields and N-nitroso compounds as well as infections may also increase the risk of developing a brain tumor. However, previous exposure to high-dose ionizing radiation is the only proven risk factor for these neoplasms [12,13]. Typical symptoms of CNS tumors include persistent headache, nausea, seizures, vomiting, neurocognitive symptoms and altered mental functions, such as personality changes. Other clinical symptoms in patients with brain tumors are: cognitive dysfunction, including changes in memory, orientation, attention, executive function, language abilities, personality and performance of daily activities [12].

The incidence of selected histological CNS tumor types may vary between different age groups. It has been shown that intracranial tumors in individuals aged over 65 years are almost five times more frequent when compared to populations under 20 years of age [17,18].

Despite the significant advances in diagnostic methods in modern medicine, glioblastoma remains incurable, with similar incidence rates and mortality [18]. Grade IV GBM are characterized by the poorest prognosis among all CNS tumors, while secondary and primary GBM have unfavorable outcome, with a median survival time shorter than one year after diagnosis. In addition, it was also reported that the median survival time of GBM patients is 6 months from diagnosis without treatment, whereas survival time can increase to around 15 months if patients receive treatment [19].

## 2. Diagnosis of Central Nervous System (CNS) Tumors

Imaging techniques are helpful in the diagnostic process of CNS tumors, including the initial work-up and follow-up as well as in the evaluation of treatment effectiveness in patients with these neoplasms. These methods are performed to assess tumor location and progression and identify its recurrence [12].

Despite the advances in structural and functional brain imaging techniques, patient survival rates are still not satisfactory. Unfortunately, neuroimaging methods are characterized by a lack of specificity for differentiation of brain tumors. Numerous investigations have led to insufficient improvement, thus tumor grading and typing are evaluated by the histopathological assessment of tissue samples, which requires surgical intervention [20]. Therefore, new approaches to the diagnosis and prognosis of CNS neoplasms are sorely needed, particularly for malignant tumors of the CNS. The easily available biochemical markers, useful in monitoring the disease course, differential diagnosis and planning of surgical interventions will improve the diagnosis of CNS tumors, particularly in the early stages of the disease [21,22].

In routine clinical management, it is only the measurement of concentrations of beta subunit of human chorionic gonadotropin (β-HCG) and alfa-fetoprotein (AFP) that are well-established biochemical tumor markers in the diagnosis of rare pediatric germ cell tumors. Moreover, the assessment of pituitary gland hormone levels in the diagnosis of pituitary tumors is also used [23,24]. Unfortunately, these biomarkers are typical only of specific and very rare types of CNS neoplasms. In addition, several authors have evaluated the levels of plasma neuropeptide Y (NPY), brain-derived neurotrophic factor (BDNF), glial cell line-derived neurotrophic factor (GDNF), placental growth factor (PlGF), S100B, interleukin 8 (IL-8) and glial fibrillary acidic protein (GFAP) in patients with different types of CNS tumors [21]. The authors concluded that all plasma concentrations of these indicators were correlated to patient parameters, including neuropathological diagnosis and neuroradiological features. However, only GFAP and PlGF were potentially useful as clinical biomarkers, which may support neuroradiological differentiation between diagnosis of GBM and intracerebral metastasis [21]. Thus, there are no blood biomarkers that have been well-investigated in the diagnosis of CNS neoplasms. Consequently, new biochemical tumor markers are sorely needed [23,24].

Various clinical investigations suggest the regulatory role of selected proteins such as cytokines, chemokines and their receptors in the pathogenesis of gliomas, particularly in the evasion of tumor cells from immunosurveillance, proliferation of tumor cells, transition from low to high-grade gliomas as well as angiogenesis within the tumor [25,26]. Moreover, many authors indicate the role of selected matrix metalloproteinases (MMPs) and their tissue inhibitors (TIMPs) in the development and progression of malignancies of CNS. The authors have revealed that these proteins might be used as potential biomarkers of human tumors, including CNS neoplasms. MMPs possess the ability to regulate many processes, including survival, angiogenesis, inflammation and signaling. As these proteases are able to degrade all ECM components, abnormal MMPs function may lead to pathological conditions. Accordingly, numerous studies have linked MMPs to pathogenesis in CNS. It is suggested that these enzymes have also been associated with the development and progression of CNS tumors such as malignant gliomas [27].

Remodeling of the ECM is crucial for tumor cell progression, including proliferation, angiogenesis and survival, thus an extensive body of literature links MMPs to tumor invasiveness, particularly metastasis [28]. Certain clinical investigations have reported increased expression of MMPs in brain tumors in situ and in vitro. The authors have revealed upregulation of the levels of MMP-2, -9 and all MT-MMP members in high-grade GBM when compared to lower-grade cases and non-transformed control brains [29]. The authors have indicated that the expression of MMPs and TIMPs as well as MT1-MMP are involved in many aspects of the pathophysiology of malignant gliomas [30,31]. Moreover, MMP-2, MT1- and MT2-MMP have been localized in glioma cells. The researchers concluded that MMP-2 and MT-MMPs might regulate glioma invasiveness, while MMP-9 plays a crucial role in angiogenesis within the tumor. In addition, the ability of various glioma lines to migrate across a reconstituted basement membrane in vitro is associated with enhanced MMP2 expression [32].

Quantitative research has revealed upregulated levels of other proteases such as uPA (a serine protease) and its receptor uPAR as well as cathepsin B (a cysteine protease) in CNS tumors [33,34]. However, ADAMs (transmembrane and secreted proteases) are unique among cell-surface proteins because of both adhesive and proteolytic activities. Some authors believe that these proteins might be promising biochemical markers involved in the pathogenesis of CNS tumors. Therefore, the objective of this review is to acquaint the reader with the biology of ADAM family members, particularly their significance in the pathogenesis of CNS tumors, especially the most frequent and deadly type–gliomas. Moreover, we will highlight the utility of ADAMs as potential biochemical biomarkers in the diagnosis of these malignancies and patient prognosis.

## 3. A Disintegrin and Metalloproteinase (ADAM)—General Information

**Figure 1:**
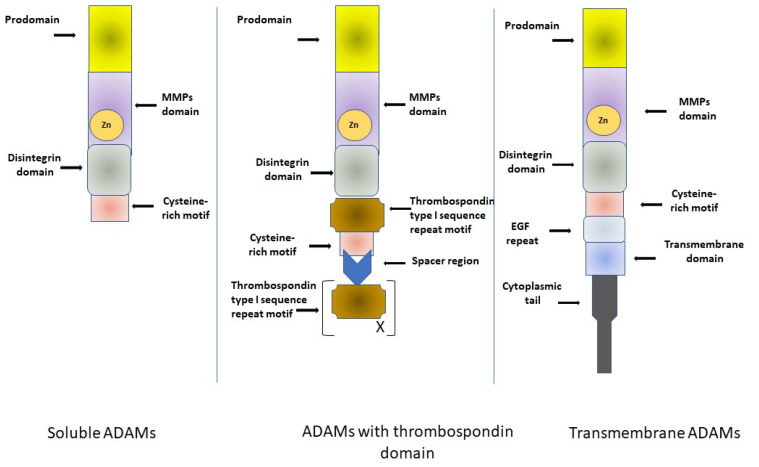
Comparative structure of a disintegrin and metalloproteinase ADAMs family members [27,35,36,37,38]. EGF, epidermal growth factor; MMPs, matrix metalloproteinases.

A Disintegrin and Metalloproteinases (ADAMs), also known as disintegrin, cysteine-rich (MDC) proteins, belong to the superfamily of zinc-dependent metalloproteinases. They are able to regulate the shedding of membrane-bound proteins, growth factors, cytokines, ligands and receptors. The structure of most ADAMs consists of a prodomain, a MMPs region, a disintegrin domain, a cysteine-rich motif, epidermal-growth-factor (EGF) repeats, a transmembrane module and a cytoplasmic tail [27,35,36,37]. Thus, these proteins are unique among cell-surface proteins because of both adhesive and proteolytic activities. Furthermore, the cysteine-rich region and EGF repeats are believed to mediate cell fusion or the interaction of ADAMs with other molecules [38,39,40]. Most ADAMs are transmembrane proteins. However, some ADAMs, e.g., ADAM 11, -12, -17 and -28 have the ability to generate a soluble, secreted protein. Around half of the ADAM family proteins have a metalloproteinase domain that contains the catalytic site consensus sequence, and in these proteins this domain possesses other functions such as allowing protein–protein interactions [27,35]. A Disintegrin and metalloproteinase with thrombospondin motifs (ADAMTS) is a family of secreted enzymes closely related to ADAMs since they have a pro-domain, and a metalloproteinase, disintegrin and cysteine-rich domain. ADAMTS have characteristic thrombospondin motifs instead of a transmembrane domain [27,35,36,37]. A comparative structure of a disintegrin and metalloproteinase ADAMs family members is presented in Figure 1.

**Figure 2:**
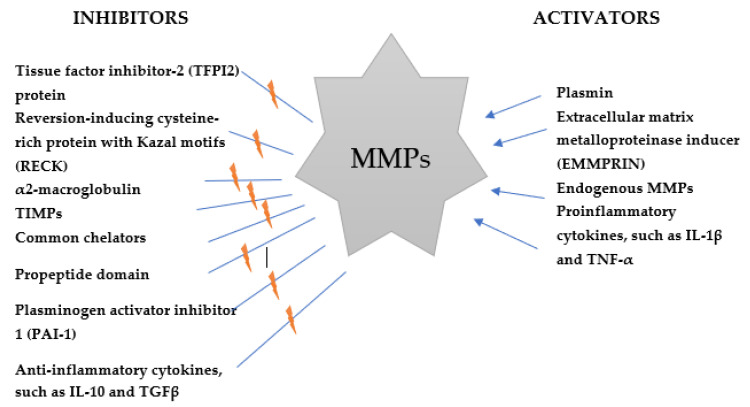
Selected inhibitors and activators of matrix metalloproteinases (MMPs).

Biological functions of ADAM family proteins include cell adhesion, sperm–egg fusion (e.g., ADAM 1, -2), ectodomain shedding of cell-surface proteins (ADAM9, -10 and -17), myoblast fusion (ADAM12) [27,36]. Therefore, these enzymes demonstrate several abilities and participate in multiple processes, including cell proliferation and migration, angiogenesis, apoptosis, tissue repair, wound healing and survival. ADAMs are involved in pathological conditions such as cardiovascular and malignant diseases as well as CNS disorders, including brain tumors [41,42]. The activity of ADAMs and ADAMTS is mainly controlled by endogenous inhibitors such as tissue inhibitors of metalloproteinases (TIMPs). Selected inhibitors and activators of metalloproteinases are presented in Figure 2. The members of the ADAM family are mostly activated by proprotein convertases. However, these proteins may also be regulated by G-protein coupled receptors (GPCRs) agonists, Ca2^+^ ionophores and protein kinase C activators. Some clinical investigations have proven that agonist-bound GPCRs are able to activate numerous MMPs, such as matrix metalloproteinase-3 (MMP3) and both gelatinases–matrix metalloproteinase-2 (MMP2) and matrix metaloproteinase-9 (MMP-9), as well as various members of the ADAMs family, including ADAM10, ADAM15 and ADAM17 [43,44,45,46]. It has been demonstrated that GPCRs bind ligands from the ECM or plasma membrane of adjacent cells, causing conveying cell–cell or cell–matrix interactions [47]. The activation of a receptor thorough binding with a ligand leads to changes in cellular processes, including cell migration, proliferation, neoangiogenesis and invasion of GBM [48]. Moreover, GPCR could be targeted for GBM therapies because of their role in controlling tumor pathogenesis through molecular interactions with oncogenic signaling pathways [48]. In addition, several ADAMs are kept in an inactive state via the interaction of a cysteine residue with zinc at the propeptide domain in the metalloproteinase module. Thus, similarly to MMPs, ADAMs is activated by the cysteine switch pathway, which interrupt the interaction of cysteine–zinc to show the catalytic site. The levels of ADAMs are also regulated at the transcriptional level. However, understanding of the physiological inhibitors of ADAMs is limited. Some investigations have revealed that only TIMP-3 inhibits the crystal structure of the protease domain of human ADAM17. Conversely, ADAM10 is inhibited by TIMP1 and -3, and by hydroxamates [33,49]. The authors conclude that TIMP3 is an inhibitor of several ADAM family members, including the ADAMTSs. However, little is known of other specific, endogenous non-TIMP ADAM inhibitors, thus further research is necessary [27,35].

## 4. A Disintegrin and Metalloproteinase (ADAM) in Brain Tumors

Despite being less widely studied than the remaining proteases such as MMPs, ADAM and ADAMTS are thought to be involved in the invasion of CNS tumors via their increased capacity to degrade the ECM [19]. The role of ADAMs in the biology of brain tumors remains to be defined [50]. Over 17 members of the ADAMs family are normally expressed in adult CNS–ADAM22 and -23 are expressed predominantly in the brain [51], while the expression of six ADAMs (ADAM9, -10, -12, -15, -17 and -19) is widespread. It has been established that ADAM17 is localized in astrocytes and endothelial cells in adult human brain [52], whereas ADAM8 has been detected in neurons and oligodendrocytes in the uninjured CNS of adult rats [53]. Thus, in our review we focus our discussion on selected ADAMs, such as ADAM8, -12, -15, -17 and -19 as well as ADMATS4 and 5, as several lines of evidence indicate their importance in malignant CNS tumors [52,54] (Table 1).

A disintegrin and metalloproteinase-12 (ADAM12) is a cell-surface glycoprotein containing metalloprotease, disintegrin, cysteine-rich and EGF-like domains which possess cell-binding and metalloprotease properties. ADAM12 is able to release of the extracellular parts of membrane-bound proteins, including glycoprotein non-metastatic melanoma protein B and basigin, heparin-binding-EGF, insulin-like growth factor 2-binding proteins 3 and 5, and oxytocinase [65,66,67,68]. It is reported that ADAM12 is predominantly localized in white and gray matter oligodendrocytes of rat and human CNS. In addition, in humans, ADAM12 occurs in two variants: membrane-anchored (ADAM12M) and secreted (ADAM12S) [69]. A study by Kodama et al. [55] using real-time quantitative polymerase chain reaction (RT-PCR) and the immunohistochemical method indicated that ADAM12M is highly expressed in human glioblastomas in comparison to non-neoplastic brain tissues, low-grade, anaplastic astrocytic tumors and intracranial neurinomas. In addition, in situ hybridization analysis demonstrated that glioblastoma cells were responsible for gene expression, while ADAM12M was mainly localized on the glioblastoma cells membranes, what was reported using immunohistochemical technique. Moreover, there was a direct correlation between mRNA expression of ADAM12M and the proliferative activity of gliomas. The authors confirmed that ADAM12M is highly expressed in human glioblastomas and indicated its role in the prominent proliferation of glioblastomas through shedding of heparin-binding EGF [55]. Interestingly, it was revealed that the signaling of EGF through the EGF receptor (EGFR) is implicated in the proliferative mechanism of glioblastomas [70]. A study by Kanakis et al. [57], which was performed using immunohistochemistry and RT-PCR methods, demonstrated the highest expression of ADAM12 in low-grade tumors. A study by Cesarini et al. [71] found that ADAM12 is also involved in the migration and invasion of glioblastoma cells. The authors reported that cellular immunostainings for ADAM12 and ADAM12 mRNA decreased with a higher histological grade of the tumor. The researchers concluded that ADAM12 might be a valuable diagnostic marker for several brain tumors [56,57].

A number of clinical investigations have shown that poor prognosis of patients with gliomas is caused by the aggressiveness of the tumor with a high probability of generating multiple metastatic growth lesions [36]. Some cell populations with an enhanced ability to self-renew and proliferate are considered to be the source of tumor development. These precursors, known as glioma stem cells or brain tumor initiating cells (BTICs), are believed to play a crucial role in the invasion and recurrence of multiple foci [72,73]. The expression of a disintegrin and metalloproteinase-9 (ADAM9) gene was significantly elevated in different BTIC lines [40]. Wildeboer et al. [54] investigated the levels of 12 cerebrally expressed ADAM genes in human primary brain tumors using RT-PCR and revealed that the mRNAs expression of five ADAMs–8, 12, 15, 17 and 19–were significantly upregulated. In addition, ADAM8 and ADAM19 proteins were located in neoplastic cells as well as in some tumors in the endothelia of blood vessels. Moreover, the authors, using specific peptide substrates for ADAM8 and ADAM19, indicated that these proteases exert increased proteolytic activity in tumors with highest expression levels [54]. Additionally, the protease activities and expression levels of ADAM8 and ADAM19 correlated with invasive activity of glioma cells, which suggests that these proteins might play a significant role in tumor invasion and may be associated with unfavorable survival [54]. Therefore, the researchers concluded that further research on ADAMs as potential targets in the therapy of primary brain tumors is sorely needed [54]. Moreover, a study by Fan et al. [58] examined the association between ADAM9 expression in lower-grade glioma (LGG) and glioblastoma (GBM) patients as well as progression-free survival and overall survival. The study demonstrated that GBM patients had significantly higher expression of ADAM9 in comparison to LGG patients, while among LGG patients, aggressive astrocytic tumors displayed significantly higher ADAM9 expression than oligodendroglial tumors [58]. In addition, elevated expression of this enzyme correlated with poor clinical outcomes in LGG patients, whereas multivariate analysis indicated that ADAM9 expression is proven to be an independent marker of poor survival [58]. The authors concluded that ADAM9 mRNA expression is associated with tumor grade and histological type in gliomas and may be used as an independent prognostic factor, particularly for survival in LGG patients [58].

A disintegrin and metalloproteinase-17 (ADAM17) has been demonstrated to be involved in proteolytic ectodomain shedding of several cytokines and membrane-bound growth factors. The elevated expression and/or activity of this protein has also been investigated in CNS tumors [59,74,75,76,77]. Thus, the authors investigated whether ADAM17 contributes to glioma progression and assessed its role in the invasion and proliferation of human glioma cells in vitro and tumor growth in vivo. The researchers revealed that ADAM17 promotes glioma cell proliferation, invasion and angiogenesis in vitro, and glioma cell growth in an animal model [74]. Moreover, a study by Chen et al. [75] determined that ADAM17 may play a role in promoting glioblastoma stem cell (GSC) invasion. It was demonstrated that CD133+ cells were found in human primary glioma tissues, which may suggest their significance in glioma invasiveness [75]. Using immunofluorescence staining the authors indicated that elevated expression of ADAM17 at the invasive front was associated with the presence of CD133+ GSCs in human glioblastoma via activation of the EGFR/PI3K/AKT signaling pathway (EGFR ligand-binding induces receptor self-dimerization, autophosphorylation and subsequently activates downstream PI3K/AKT) [76]. Therefore, these findings suggest that ADAM17 is able to mediate the invasive process in glioblastoma stem cell and may be used as therapeutic target for treatment of glioma [77]. A similar study was performed by Wu et al. [59] who showed that ADAM17 expression was significantly associated with the WHO histological grade of the glioma. Thus, the levels of this protein increased with tumor grade and were upregulated in high-grade glioma tissues compared to low-grade and normal brain tissues. In addition, the survival rate of patients with ADAM17-positive tumors was lower compared to individuals with ADAM17-negative tumors. These findings suggest that the overexpression of ADAM17 is correlated with a high tumor grade and a poor prognosis of glioma patients and may be a potential diagnostic and therapeutic target [59].

A study by Qu et al. [60] using RT-PCR, Western blot analysis and immunohistochemistry assessed mRNA and protein expression levels of a disintegrin and metalloproteinase-10 (ADAM10) in glioma cells. The expression of ADAM10 was significantly elevated in low-grade glioma samples compared with the control group (meningioma), while being further increased in high-grade glioma samples, which may suggest that mRNA and protein expression levels of ADAM10 are closely associated with glioma grade in a malignancy-dependent pattern [60]. In addition, ADAM10 protein was found on the tumor cell membrane and blood vessel walls within tumor tissues, which was assessed using immunohistochemical analysis. The authors concluded that ADAM10 expression correlates with the grade of malignancy in human glioma and may indicate an important biological role of ADAM10 in glioma growth and development, particularly the invasiveness of gliomas as well as in peritumoral edema formation [60]. Moreover, the most recent publications demonstrate that ADAM10 inhibitors might be used in the treatment of glioma patients [78]. ADAM10 is able to cleave numerous substrates including e.g., programmed death-ligand 1 (PD-L1), EGFR/HER ligands or the MHC class I-related molecules (MIC-A, and MIC-B) expressed by malignant cells. The authors have proposed several mechanisms involved in the promotion of GBM by ADAM10. Neuroligin-3 (NLGN3) levels have been linked to high-grade GBM, stimulating proliferation of GBM cells via e.g., prooncogenic gene expression through focal adhesion kinase (FAK) and synapse-related gene expression [79,80]. Another pathway implicates the cleavage of N-cadherin by ADAM10′s in cell migration and metastasis, thus treatment of GBM cell lines with an antibody to inhibit ADAM10 decreased tumor growth and migration [81]. In addition, natural killer cells (NKs) have been proven to stimulate an immune response against GBM and these cells are associated with improved prognosis of glioma patients [82]. NK cells recognize GBM by binding ligands to the NK cell-activating (NKG2D) receptors that are expressed in malignant cells. The authors concluded that ADAM10 seems to be a therapeutic candidate to target GBM due to its ability to cleave multiple substrates implicated in disease progression [78].

Other studies have examined the elevated expression of ADAMTS4 and ADAMTS5 (a disintegrin and metalloproteinase with thrombospondin motifs 4 and 5) in glioma samples on the mRNA and protein levels [61,83]. Using RT-PCR the authors proved that selected gliomas express ADAMTS4 and -5 when grown in situ, while GBM samples demonstrated the highest expression of these proteins, implicating these factors in malignancy [61]. In addition, the levels of ADAMTS4 and 5 were more elevated in solid GBM tumor samples in situ than in cultivated glioblastoma cells. The authors concluded that these differences are not due to the production of ADAMTS4 and 5 by other cells in solid tumors, but might be a result of their upregulation by growth factors and cytokines produced by tumor cells [61,62]. Interestingly, immunohistochemical double-labeling has revealed that the expression of these proteins is restricted to GBM and astroglial cells, whereas the discrepancies in ADAMTS levels between sample sources are associated with the nature of their regulation in vivo. The authors concluded that ADAMTS4 and 5 are upregulated in proliferating glioblastoma cells and these proteases may promote their invasive potential [61]. Moreover, ADAMTS4 mRNA is expressed by glioma cell lines with invasive properties and the cleavage of brevican by this enzyme is closely associated with the invasiveness of brain tumors [61,62,83,84,85,86]. Interestingly, inhibitors of ADAMTS could potentially serve to limit glioma migration [83].

A study by Dunn et al. reported that the expression of ADAMTS8 is downregulated in brain tumors in comparison to a normal brain [76]. The researchers indicated that mRNA levels were reduced at least twofold in 100% tumors and glioma cell lines tested. Moreover, immunohistochemical analysis revealed decreased levels of ADAMTS8 in 77% of brain tumors tested compared to non-neoplastic tissue, while this protein was undetectable in 67% of tumors in Western blot method. These findings, as well as studies by other authors, suggest that ADAMTS8 is downregulated in cancers and demonstrate its role in brain tumorigenesis [63,64,87].

## 5. Conclusions

Although it is important to develop new treatment modalities, a more comprehensive knowledge of CNS tumors, particularly the pathogenesis of GBM, is crucial to improve the diagnostic process of these neoplasms. In contemporary medicine, no curative therapy is known for the most common malignant brain tumors–gliomas, and there is no therapeutic approach that significantly prolongs the life of patients with this malignancy. Therefore, the biology of CNS tumors must be studied to improve the diagnosis of these tumors and patient prognosis as well as to define potential targets for therapeutic approaches. Studies performed on malignant gliomas have achieved considerable progress, but to a large degree the disease is still incurable. The median survival time for patients with high-grade gliomas is no more than 15 months, mostly due to their aggressiveness with a high probability of generating multiple metastatic growth lesions.

The significance of selected MMPs and TIMPs in CNS neoplasms has been studied extensively in CNS tumors. However, ADAMs are unique among cell-surface proteins because of both adhesive and proteolytic activities. Therefore, in our review we have summarized the current body of knowledge concerning the role of selected ADAMs and ADAMTS in the development of CNS neoplasms. We have also highlighted several ADAM proteins associated with CNS tumors, particularly the most common and deadly type of CNS tumors–gliomas.

A number of clinical investigations suggest that the proteins of the ADAMs family are involved in different features of glioma behavior, including migration of cancer cells and ectodomain shedding of immune receptors or/and various growth factors. The overexpression of several ADAMs in selected brain tumors, particularly glioblastomas has been described. It has been proven that ADAM12, ADAMTS4 and 5 are implicated in the proliferation and invasion of glioma cells. ADAM8 and ADAM19 are correlated with the invasive activity of glioma cells and may be associated with unfavorable survival, while ADAM9 is associated with tumor grade and histological type in gliomas and might be an independent prognostic factor, especially in LGG patients. In addition, ADAM17 is correlated with a high tumor grade and poor prognosis in patients with glioma and may be a potential diagnostic and therapeutic target, whereas ADAM10 is associated with glioma grade in glioma growth and development, particularly the invasiveness of the glioma. ADAMTS8 is downregulated in brain cancers and may play a role in brain tumorigenesis.

In conclusion, several ADAMs and ADAMTS can serve as potential diagnostic and prognostic biomarkers as well as therapeutic targets for CNS tumors. However, there is a need for research into novel approaches exploring the potential usefulness of these enzymes in the diagnosis of CNS tumors and prognosis of patients with these neoplasms via elevated changes in the profile of these proteins in different histological types of the tumor and stages of the disease. Although enhanced levels of selected ADAMs and ADAMTS have been correlated with CNS tumor progression, the regulatory mechanisms responsible for this multistep process are still poorly understood. Therefore, future investigations need to be evaluated before these proteins can be used as biomarkers of specific types of CNS neoplasms.

## Figures and Tables

**Table 1:** The significance of selected ADAM family members as potential biomarkers of CNS tumors.

ADAMs/ADAMTS	Method	Results	Literature
ADAM12	RT-PCR Immunohistochemistry	Markedly enhanced expression in brain tumors.	[55]
	RT-PCR Gelatin zymography Immunoblotting	Involved in the migration and invasion of glioblastoma cells.	[56]
	RT-PCR Immunohistochemistry	Expression decreased with higher histological grade of the tumor. Helpful tool in the diagnosis of brain tumor.	[57]
ADAM8, -12, -15, -17, and -19	RT-PCR Immunoblotting Immunohistochemistry	Elevated expression of ADAM8 and ADAM19 and protease activities correlate with invasive activity of glioma cells. A significant role of ADAM8 and ADAM19 in tumor invasion.	[54]
ADAM9	Read Mapping and Expression Analysis of RefSeq Genes	mRNA expression is associated with tumor grade and histological type in gliomas and may be used as an independent prognostic factor, particularly in LGG patients.	[58]
ADAM17	RT-PCR Western blot analysis Immunohistochemistry	Overexpression is correlated with a high tumor grade and poor prognosis in patients with glioma. Potential diagnostic and therapeutic target.	[59]
ADAM10	RT-PCR Western blot analysis Immunohistochemistry	Expression is closely associated with glioma grade in a malignancy-dependent pattern. Important biological role in glioma growth and development, particularly the invasiveness of gliomas.	[60]
ADAMTS4, -5	RT-PCR	Expression is upregulated in proliferating glioblastoma cells and these proteases may promote their invasive potential.	[61,62]
ADAMTS8	RT-PCR Western blot analysis Immunohistochemistry	Expression of ADAMTS8 is downregulated in brain tumors in comparison to normal brain. A role in brain tumorigenesis.	[63,64]

## Data Availability

Not applicable.

## References

[1] Louis D.N., Ohgaki H., Wiestler O.D., Cavenee W.K., Burger P.C., Jouvet A., Scheithauer B.W., Kleihues P. (2007). The 2007 WHO classification of tumours of the central nervous system. Acta Neuropathol..

[2] Louis D.N., Perry A., Reifenberger G., von Deimling A., Figarella-Branger D., Cavenee W.K., Ohgaki H., Wiestler O.D., Kleihues P., Ellison D.W. (2016). The 2016 World Health Organization Classification of Tumors of the Central Nervous System: A summary. Acta Neuropathol..

[3] Rousseau A., Mokhtari K., Duyckaerts C. (2008). The 2007 WHO classification of tumors of the central nervous system—what has changed?. Curr. Opin. Neurol..

[4] Shaw E.G., Berkey B., Coons S.W., Bullard D., Brachman D., Buckner J.C., Stelzer K.J., Barger G.R., Brown P.D., Gilbert M.R. (2008). Recurrence following neurosurgeon-determined gross-total resection of adult supratentorial low-grade glioma: Results of a prospective clinical trial. J. Neurosurg..

[5] Chaichana K.L., McGirt M.J., Laterra J., Olivi A., Quiñones-Hinojosa A. (2010). Recurrence and malignant degeneration after resection of adult hemispheric low-grade gliomas. J. Neurosurg..

[6] Ostrom Q.T., Gittleman H., Truitt G., Boscia A., Kruchko C., Barnholtz-Sloan J.S. (2018). CBTRUS Statistical Report: Primary Brain and Other Central Nervous System Tumors Diagnosed in the United States in 2011–2015. Neuro Oncol..

[7] Ohgaki H., Kleihues P. (2007). Genetic pathways to primary and secondary glioblastoma. Am. J. Pathol..

[8] Nakamura M., Shimada K., Ishida E., Higuchi T., Nakase H., Sakaki T., Konishi N. (2007). Molecular pathogenesis of pediatric astrocytic tumors. Neuro Oncol..

[9] Fujisawa H., Reis R.M., Nakamura M., Colella S., Yonekawa Y., Kleihues P., Ohgaki H. (2000). Loss of heterozygosity on chromosome 10 is more extensive in primary (de novo) than in secondary glioblastomas. Lab. Investig..

[10] Ohgaki H., Kleihues P. (2013). The definition of primary and secondary glioblastoma. Clin. Cancer Res..

[11] Crespo I., Vital A.L., Gonzalez-Tablas M., del Carmen Patino M., Otero A., Lopes M.C., de Oliveira C., Domingues P., Orfao A., Tabernero M.D. (2015). Molecular and Genomic Alterations in Glioblastoma Multiforme. Am. J. Pathol..

[12] Chandana S.R., Movva S., Arora M., Singh T. (2008). Primary brain tumors in adults. Am. Fam. Physician.

[13] Fisher J.L., Schwartzbaum J.A., Wrensch M., Berger M.S. (2006). Evaluation of epidemiologic evidence for primary adult brain tumor risk factors using evidence-based medicine. Prog. Neurol. Surg..

[14] Keyvani-Ghamsari S., Khorsandi K., Rasul A., Zaman M.K. (2021). Current understanding of epigenetics mechanism as a novel target in reducing cancer stem cells resistance. Clin. Epigenetics.

[15] Burgess R., Jenkins R., Zhang Z. (2008). Epigenetic changes in gliomas. Cancer Biol. Ther..

[16] Kim Y.Z. (2014). Altered Histone Modifications in Gliomas. Brain Tumor Res. Treat..

[17] GBD 2016 Brain and Other CNS Cancer Collaborators (2019). Global, regional, and national burden of brain and other CNS cancer, 1990–2016: A systematic analysis for the Global Burden of Disease Study 2016. Lancet Neurol..

[18] Lin L., Yan L., Liu Y., Yuan F., Li H., Ni J. (2019). Incidence and death in 29 cancer groups in 2017 and trend analysis from 1990 to 2017 from the Global Burden of Disease Study. J. Hematol. Oncol..

[19] Hatoum A., Mohammed R., Zakieh O. (2019). The unique invasiveness of glioblastoma and possible drug targets on extracellular matrix. Cancer Manag. Res..

[20] van den Bent M.J., Vogelbaum M.A., Wen P.Y., Macdonald D.R., Chang S.M. (2009). End point assessment in gliomas: Novel treatments limit usefulness of classical Macdonald’s Criteria. J. Clin. Oncol..

[21] Ilhan-Mutlu A., Wagner L., Widhalm G., Wöhrer A., Bartsch S., Czech T., Heinal H., leutmezer F., Prayer D., Marosi C. (2013). Exploratory investigation of eight circulating plasma markers in brain tumor patients. Neurosurg. Rev..

[22] Iwamoto F.M., Hottinger A.F., Karimi S., Riedel E., Dantis J., Jahdi M., Panageas K.S., Lassman A.B., Abrey L.E., Fleisher M. (2011). Longitudinal prospective study of matrix metalloproteinase-9 as a serum marker in gliomas. J. Neurooncol..

[23] Jorsal T., Rørth M. (2012). Intracranial germ cell tumours. A review with special reference to endocrine manifestations. Acta Oncol..

[24] Englund A.T., Geffner M.E., Nagel R.A., Lippe B.M., Braunstein G.D. (1991). Pediatric germ cell and human chorionic gonadotropin-producing tumors. Clinical and laboratory features. Am. J. Dis. Child..

[25] Lukaszewicz-Zając M., Mroczko B., Kornhuber J., Lewczuk P. (2014). Matrix metalloproteinases (MMPs) and their tissue inhibitors (TIMPs) in the tumors of central nervous system (CNS). J. Neural Transm..

[26] Groblewska M., Litman-Zawadzka A., Mroczko B. (2020). The Role of Selected Chemokines and Their Receptors in the Development of Gliomas. Int. J. Mol. Sci..

[27] Yong V.W., Krekoski C.A., Forsyth P.A., Bell R., Edwards D.R. (1998). Matrix metalloproteinases and diseases of the central nervous system. Trends Neurosci..

[28] McCawley L.J., Matrisian L.M. (2000). Matrix metalloproteinases: Multifunctional contributors to tumor progression. Mol. Med. Today.

[29] Lampert K., Machein U., Machein M.R., Conca W., Peter H.H., Volk B. (1998). Expression of matrix metalloproteinases and their tissue inhibitors in human brain tumors. Am. J. Pathol..

[30] Forsyth P.A., Wong H., Laing T.D., Rewcastle N.B., Morris D.G., Muzik H., Leco K.J., Johnston R.N., Brasher P.M., Sutherland G. (1999). Gelatinase-A (MMP-2), gelatinase-B (MMP-9) and membrane type matrix metalloproteinase-1 (MT1-MMP) are involved in different aspects of the pathophysiology of malignant gliomas. Br. J. Cancer.

[31] Nakada M., Kite D., Futami K., Yamashita J., Fujimoto N., Sato H., Okada Y. (2001). Roles of membrane type 1 matrix metalloproteinase and tissue inhibitor of metalloproteinases 2 in invasion and dissemination of human malignant glioma. J. Neurosurg..

[32] Uhm J.H., Dooley N.P., Villemure J.G., Yong V.W. (1996). Glioma invasion in vitro: Regulation by matrix metalloprotease-2 and protein kinase C. Clin. Exp. Metastasis.

[33] Mentlein R., Hattermann K., Held-Feindt J. (2012). Lost in disruption: Role of proteases in glioma invasion and progression. Biochim. Biophys. Acta.

[34] Rao J.S. (2003). Molecular mechanisms of glioma invasiveness: The role of proteases. Nat. Rev. Cancer.

[35] Yong V.W., Power C., Forsyth P., Edwards D.R. (2001). Metalloproteinases in biology and pathology of the nervous system. Nat. Rev. Neurosci..

[36] Haoyuan M.A., Yanshu L.I. (2020). Structure, regulatory factors and cancer-related physiological effects of ADAM9. Cell Adh. Migr..

[37] Giebeler N., Zigrino P. (2016). A Disintegrin and Metalloprotease (ADAM): Historical Overview of Their Functions. Toxins.

[38] Chute M., Jana S., Kassiri Z. (2018). Disintegrin and metalloproteinases (ADAMs and ADAM-TSs), the emerging family of proteases in heart physiology and pathology. Curr. Opin. Physiol..

[39] Schlondorff J., Blobel C.P. (1999). Metalloprotease-disintegrins: Modular proteins capable of promoting cell–cell interactions and triggering signals by protein-ectodomain shedding. J. Cell Sci..

[40] Izumi Y., Hirata M., Hasuwa H., Iwamoto R., Umata T., Miyado K., Tamai Y., Kurisaki T., Sehara-Fujisawa S.A., Ohno E. (1998). A metalloprotease-disintegrin, MDC9/meltrin-γ/ADAM9 and PKC δ are involved in TPA-induced ectodomain shedding of membrane-anchored heparinbinding EGF-like growth factor. EMBO J..

[41] Kang Q., Cao Y., Zolkiewska A. (2000). Metalloproteasedisintegrin. ADAM 12 binds to the SH3 domain of Src and activates Src tyrosine kinase in C2C12 cells. Biochem. J..

[42] Bernstein H., Keilhoff G., Dobrowolny H., Lendeckel U., Steiner J. (2020). From putative brain tumor marker to high cognitive abilities: Emerging roles of a disintegrin and metalloprotease (ADAM) 12 in the brain. J. Chem. Neuroanat..

[43] Lee M.H., Murphy G. (2004). Matrix metalloproteinases at a glance. J. Cell Sci..

[44] Le Gall S.M., Auger R., Dreux C., Mauduit P. (2003). Regulated cell surface pro-EGF ectodomain shedding is a zinc metalloprotease-dependent process. J. Biol. Chem..

[45] Murasawa S., Mori Y., Nozawa Y., Gotoh N., Shibuya M., Masaki H., Maruyama K., Tsutsumi Y., Moriguchi Y., Shibazaki Y. (1998). Angiotensin II type 1 receptor-induced extracellular signal-regulated protein kinase activation is mediated by Ca^2+^/calmodulin-dependent transactivation of epidermal growth factor receptor. Circ. Res..

[46] Göőz M., Göőz P., Luttrell L.M., Raymond J.R. (2006). 5-HT2A receptor induces ERK phosphorylation and proliferation through ADAM-17 tumor necrosis factor-alpha-converting enzyme (TACE) activation and heparin-bound epidermal growth factor-like growth factor (HB-EGF) shedding in mesangial cells. J. Biol. Chem..

[47] Cherrya A.E., Stellaa N. (2014). G protein-coupled receptors as oncogenic signals in glioma: Emerging therapeutic avenues. Neuroscience.

[48] Stephan G., Ravn-Boess N., Placantonakis D.G. (2021). Adhesion G protein-coupled receptors in glioblastoma. Neuro-Oncol. Adv..

[49] Amour A., Knight C.G., Webster A., Slocombe P.M., Stephens P.E., Knäuper V., Docherty A.J., Murphy G. (2000). The in vitro activity of ADAM-10 is inhibited by TIMP-1 and TIMP-3. FEBS Lett..

[50] Harada T., Nishie A., Torigoe K., Ikezaki K., Shono T., Maehara Y., Kuwano M., Wada M. (2000). The specific expression of three novel splice variant forms of human metalloprotease-like disintegrin-like cysteine-rich protein 2 gene in brain tissues and gliomas. Jpn. J. Cancer Res..

[51] Karkkainen I., Rybnikova E., Pelto-Huikko M., Huovila A.P. (2000). Metalloprotease-disintegrin (ADAM) genes are widely and differentially expressed in the adult CNS. Mol. Cell Neurosci..

[52] Goddard D.R., Bunning R.A.D., Woodroofe M.N. (2001). Astrocyte and endothelial cell expression of ADAM 17 (TACE) in adult human CNS. Glia.

[53] Schlomann U., Rathke-Hartlieb S., Yamamoto S., Jockusch H., Bartsch J.W. (2000). Tumor necrosis factor α induces a metalloprotease-disintegrin, ADAM8 (CD156): Implications for neuron–glia interactions during neurodegeneration. J. Neurosci..

[54] Wildeboer D., Naus S., Sang Q.S., Bartsch J.W., Pagenstecher A. (2006). Metalloproteinase disintegrins ADAM8 and ADAM19 are highly regulated in human primary brain tumors and their expression levels and activities are associated with invasiveness. J. Neuropathol. Exp. Neurol..

[55] Kodama T., Ikeda E., Okada A., Ohtsuka T., Shimoda M., Shiomi T., Yoshida K., Nakada M., Ohuchi E., Okada Y. (2004). ADAM12 is selectively overexpressed in human glioblastomas and is associated with glioblastoma cell proliferation and shedding of heparin-binding epidermal growth factor. Am. J. Pathol..

[56] Kurisaki T., Masuda A., Sudo K., Sakagami J., Higashiyama S., Matsuda Y., Nagabukuro A., Tsuji A., Nabeshima Y., Asano M. (2003). Phenotypic analysis of Meltrin alpha (ADAM12)-deficient mice: Involvement of Meltrin alpha in adipogenesis and myogenesis. Mol. Cell Biol..

[57] Kanakis D., Lendeckel U., Theodosiou P., Dobrowolny H., Mawrin C., Keilhoff G., Bukowska A., Dietzmann K., Bogerts B., Bernstein H.G. (2013). ADAM 12: A putative marker of oligodendrogliomas?. Dis. Markers.

[58] Fan X., Wang Y., Zhang C., Liu L., Yang S., Wang Y., Liu X. (2016). ADAM9 Expression Is Associate with Glioma Tumor Grade and Histological Type, and Acts as a Prognostic Factor in Lower-Grade Gliomas. Int. J. Mol. Sci..

[59] Wu B., Sha B., Wang Y., Xu W., Yu Y., Feng F., Sun C., Xia L. (2014). Diagnostic and prognostic value of a disintegrin and metalloproteinase-17 in patients with gliomas. Oncol. Lett..

[60] Qu M., Qiu B.O., Xiong W., Chen D., Wu A. (2015). Expression of a-disintegrin and metalloproteinase 10 correlates with grade of malignancy in human glioma. Oncol. Lett..

[61] Held-Feindt J., Paredes E.B., Blomer U., Seidenbecher C., Stark A.M., Mehdorn H.M., Mentlein R. (2006). Matrix-degrading proteases ADAMTS4 and ADAMTS5 (disintegrins and metalloproteinases with thrombospondin motifs 4 and 5) are expressed in human glioblastomas. Int. J. Cancer..

[62] Held-Feindt J., Mentlein R. (2002). CD70/CD27 ligand, a member of the TNF family, is expressed in human brain tumours. Int. J. Cancer.

[63] Dunn J.R., Reed J.E., du Plessis D.G., Shaw E.J., Reeves P., Gee A.L., Warnke P., Walker C. (2006). Expression of ADAMTS-8, a secreted protease with antiangiogenic properties, is downregulated in brain tumours. Br. J. Cancer.

[64] Dunn J.R., Panutsopulos D., Shaw M.W., Heighway J., Dormer R., Salmo E.N., Watson S.G., Field J.K., Liloglou T. (2004). METH-2 silencing and promoter hypermethylation in NSCLC. Br. J. Cancer.

[65] White J.M. (2003). ADAMs: Modulators of cell-cell and cell-matrix interactions. Curr. Opin. Cell Biol..

[66] Tanaka H., Shimazawa M., Kimura M., Takata M., Tsuruma K., Yamada M., Takahashi H., Hozumi I., Niwa J., Iguchi Y. (2012). The potential of GPNMB as novel neuroprotective factor in amyotrophic ateral sclerosis. Sci. Rep..

[67] Lendeckel U., Wolke C., Bernstein H.G., Keilhoff G. (2015). Effects of nitric oxide synthase deficiency on a disintegrin and metalloproteinase domain-containing protein 12 expression in mouse brain samples. Mol. Med. Rep..

[68] Albrechtsen R., Wewer Albrechtsen N.J., Gnosa S., Schwarz J., Dyrskjøt L., Kveiborg M. (2019). Identification of ADAM12 as a novel basigin sheddase. Int. J. Mol. Sci..

[69] Wever U.M., Albrechtsen R., Engvall E., Hooper N.M., Lendeckel U. (2005). ADAM12 the long and the short of it. The ADAM Family of Proteases.

[70] Kleihues P., Cavenee K., Pathology & Genetics (2000). Tumours of the Nervous System. World Health Organisation Classification of Tumours.

[71] Cesarini V., Silvestris D.A., Tassinari V., Tomaselli S., Alon S., Eisenberg E., Locatelli F., Gallo A. (2018). ADAR2/miR-589-3p axis controls glioblastoma cell migration/invasion. Nucleic Acids Res..

[72] Choi S.A., Lee J.Y., Phi J.H., Wang K.-C., Park C.-K., Park S.-H., Kim S.-K. (2014). Identification of brain tumour initiating cells using the stem cell marker aldehyde dehydrogenase. Eur. J. Cancer.

[73] Modrek A.S., Bayin N.S., Placantonakis D.G. (2014). Brain stem cells as the cell of origin in glioma. World J. Stem Cells.

[74] Zheng X., Jiang F., Katakowski M., Lu Y., Chopp M. (2012). ADAM17 promotes glioma cell malignant phenotype. Mol. Carcinog..

[75] Chen X., Chen L., Chen J., Hu W., Gao H., Xie B., Wang X., Yin Z., Li S., Wang X. (2013). ADAM17 promotes U87 glioblastoma stem cell migration and invasion. Brain Res..

[76] Feng Y., Dai X., Li X., Wang H., Liu J., Zhang J., Du Y., Xia L. (2012). EGF signalling pathway regulates colon cancer stem cell proliferation and apoptosis. Cell Prolif..

[77] Calligaris M., Cuffaro D., Bonelli S., Spanò D.P., Rossello A., Nuti E., Scilabra S.D. (2021). Strategies to Target ADAM17 in Disease: From Its Discovery to the iRhom Revolution. Molecules.

[78] Smith T.M., Tharakan A., Martin R.K. (2000). Targeting ADAM10 in Cancer and Autoimmunity. Front. Immunol..

[79] Venkatesh H.S., Tam L.T., Woo P.J., Lennon J., Nagaraja S., Gillespie S.M., Ni J., Duveau D.Y., Morris P.J., Zhao J.J. (2017). Targeting neuronal activity-regulated neuroligin-3 dependency in high-grade glioma. Nature.

[80] Liu R., Qin X.-P., Zhuang Y., Zhang Y., Liao H.-B., Tang J.-C., Pan M.-X., Zeng F.-F., Lei Y., Lei R.-X. (2018). Glioblastoma recurrence correlates with NLGN3 levels. Cancer Med..

[81] Kohutek Z.A., Charles G., Redpath G.T., Hussaini I.M. (2009). ADAM-10-mediated N-Cadherin cleavage is protein kinase C-alpha dependent and promotes glioblastoma cell migration. J. Neurosci..

[82] Wang J., Matosevic S. (2019). NT5E/CD73 as correlative factor of patient survival and natural killer cell infiltration in glioblastoma. J. Clin. Med..

[83] Gottschall P.E., Howell M.D. (2015). ADAMTS expression and function in central nervous system injury and disorders. Matrix Biol..

[84] Matthews R.T., Gary S.C., Zerillo C., Pratta M., Solomon K., Arner E.C., Hockfield S. (2000). Brain-enriched hyaluronan binding (BEHAB)/ brevican cleavage in a glioma cell line is mediated by a disintegrin and metalloproteinase with thrombospondin motifs (ADAMTS) family member. J. Biol. Chem..

[85] Viapiano M.S., Hockfield S., Matthews R.T. (2008). BEHAB/brevican requires ADAMTS-mediated proteolytic cleavage to promote glioma invasion. J. Neuro-Oncol..

[86] Nakada M., Miyamori H., Kita D., Takahashi T., Yamashita J., Sato H., Miura R., Yamaguchi Y., Okada Y. (2005). Human glioblastomas overexpress ADAMTS-5 that degrades brevican. Acta Neuropathol..

[87] Porter S., Scott S.D., Sassoon E.M., Williams M.R., Jones J.L., Girling A.C., Ball R.Y., Edwards D.R. (2004). Dysregulated expression of adamalysin-thrombospondin genes in human breast carcinoma. Clin. Cancer Res..

